# Development of Chitosan Polymer Membranes with Potential Use in Filtration Processes

**DOI:** 10.3390/membranes16010031

**Published:** 2026-01-04

**Authors:** Ana Luisa Aguilar-Ruiz, Tomás Jesús Madera-Santana, Reyna G. Sánchez-Duarte, Yedidia Villegas-Peralta, Ana Alejandra Aguilar-Ruiz, Víctor Manuel Orozco-Carmona

**Affiliations:** 1 Departamento de Ciencias del Agua y Medio Ambiente, Instituto Tecnológico de Sonora, 5 de Febrero 818 Sur, Ciudad Obregón 85000, Sonora, Mexico; ana.aguilar@itson.edu.mx (A.L.A.-R.); ana.aguilar111695@potros.itson.edu.mx (A.A.A.-R.); 2 Coordinación de Tecnología de Alimentos de Origen Vegetal, Centro de Investigación en Alimentación y Desarrollo, A.C. Carr. Gustavo E. Astiazarán No. 46, Hermosillo 83304, Sonora, Mexico; madera@ciad.mx; 3 Centro de Investigación en Materiales Avanzados S.C., Ave. Miguel de Cervantes 120, Complejo Industrial Chihuahua, Chihuahua 31136, Chihuahua, Mexico; victor.orozco@cimav.edu.mx

**Keywords:** membranes, biopolymer, crosslinkers, chitosan, filtration process

## Abstract

Polymeric membranes based on chitosan (Cs) were extracted from shrimp shells and evaluated. These membranes were modified using polyethylene glycol (PEG), polyvinylpyrrolidone (PVP), and glycerol (Gly) and crosslinked with glutaraldehyde (GA) to examine their suitability for water filtration processes. The Cs exhibited high purity, a total nitrogen content of 6.49%, and an average molecular weight of 456 kDa, all of which are suitable for membrane formation. Four membranes (Cs-GA, B2: Cs-PEG, B5: Cs-PEG-PVP, and B7: Cs-Gly) were characterized by means of FTIR, SEM, AFM, thickness, contact angle, tensile testing, TGA, DSC, and filtration with distilled water at 4.83 bar. B2 and B5 showed thicknesses of 207 and 190 μm and contact angles of 56.7° and 58.9°, lower than that of Cs-GA (89.4°). In filtration, B2 achieved a flux of 2222.70 LMH, a permeance of 460.19 LMH·bar^−1^, and a hydraulic resistance of 8.79 × 10^11^ m^−1^, while Cs-GA, B5, and B7 exhibited fluxes of 24.10, 40.43, and 24.77 LMH, respectively, permeances of 9.75, 8.37, and 5.13 LMH·bar^−1^, and hydraulic resistances of 4.15 × 10^13^, 4.83 × 10^13^, and 7.89 × 10^13^ m^−1^, in the same order. Overall, membranes B2 and B5 are recognized as the most promising for water filtration under pressured operating conditions.

## 1. Introduction

Water is an essential resource for human survival and development, as it is used for direct consumption, hygiene, and sanitation. Approximately 70% of the Earth’s surface is covered by water. This total volume is 97.5% saltwater found in seas and oceans, while only 2.5% is available freshwater; approximately 79% is frozen in glaciers, and about 30% exist as groundwater. Only 1% of the total freshwater, which is equivalent to 0.025% of all of the water on the planet, is available in rivers, lakes, and other surface water bodies that can be accessed for human consumption [1]. It is worth noting that most of this freshwater is used for agriculture and livestock farming, followed by industrial and energy generation sectors [2,3]. However, the repeated use of water bodies as final receptacles for various waste streams has led to a significant decline in water quality, compromising its availability and sustainable use [4].

Water quality degradation is mainly due to the persistent discharge of chemical and biological pollutants into different aquatic systems, as well as the increasingly limited availability of the resource, attributed to population growth, accelerated urbanization, industrial expansion, and the progressive decrease in rainfall regimes observed in various regions of the world as a climate change consequence [5,6]. Water scarcity is one of the most significant problems globally; up to 40% of the world’s population faces severe scarcity [3]. This phenomenon has motivated numerous research projects aimed at finding alternatives that efficiently and sustainably improve water quality from various sources, enabling its reuse or allocation to different uses [7]. Alternatives to mitigate water scarcity include two main categories: thermal and membrane-based processes. These separation technologies stand out not only for their efficiency but also for their promotion of an environmental sustainability perspective [8,9]. In recent decades, desalination processes have gained significant importance, enabling the use of 97% of previously underutilized seawater to meet human, agricultural, and industrial demands. Currently, reverse osmosis (RO) is one of the most widely used technologies for water desalination and producing potable water from rivers, lakes, wells, and other sources [10].

The reverse osmosis (RO) process consists of a system that forces a fluid under pressure through a semi-permeable membrane. These systems are notable for their high efficiency, compact design, and operational flexibility. However, RO has limitations related to its high energy requirements and susceptibility to membrane fouling/biofouling, factors that significantly reduce treated water recovery efficiency and increase operating expenses from 25 to 50% [11]. The most commonly used materials for membrane fabrication in separation processes, such as desalination, are resistant, semipermeable polymers, including polyamide and, to a lesser extent, cellulose acetate [12]. However, a growing interest has emerged in using biopolymers as base materials for membrane production. Chitosan (Cs) has gained significant attention due to its biocompatibility, antimicrobial properties, and extensive chemical functionality, which enable modifying its polymer chain. Nevertheless, Cs films exhibit mechanical limitations and a marked hydrophilic tendency, which compromise the balance between flow, selectivity, and long-term stability [13].

Chitosan (Cs) is a natural polysaccharide containing primary amino and hydroxyl groups along its chain, which confer high chemical reactivity. Furthermore, it is insoluble in most organic solvents, expanding its potential applications. These characteristics have made chitosan a versatile base for developing new materials—for example, cross-linked membranes—in biotechnology, biomedicine, agriculture, and filtration processes [14,15].

To overcome Cs limitations, various polymer grafts can be incorporated, and crosslinking agents can be used to modify the polymer chain density, porosity, surface wettability, and mechanical properties of the material. Hydrophilic polymers such as polyethylene glycol (PEG), polyvinylpyrrolidone (PVP), and glycerol (Gly) act as pore formers or plasticizers by improving chain compatibility and mobility, thereby promoting controlled porosity and affinity. On the other hand, adjusting the proportions of crosslinkers, such as glutaraldehyde (GA), strengthens the Cs polymer network and increases its chemical stability [16,17,18].

In the present work, Cs-based membranes were prepared by incorporating PEG, PVP, and Gly with GA used as a crosslinking agent. FTIR was used to characterize chitosan; molecular weight was determined by means of capillary viscometry, and total nitrogen, moisture, and ash content were measured. The impact of processing on membrane morphology was examined using SEM and AFM analyses. Thickness, wettability (contact angle), and mechanical and thermal behavior were also evaluated using TGA and DSC analyses. Finally, membrane performance was assessed in filtration tests using permeate flux (*J*), permeance (L_p_), and hydraulic resistance (R).

## 2. Materials and Methods

The chemicals used in the present investigation were as follows: poly(ethylene glycol) with a molecular weight of 20 kDa, obtained from Merck (Darmstadt, Germany); polyvinylpyrrolidone with an approximate molecular weight of 29 kDa; glutaraldehyde solution (25%, Mw = 100.12 g·mol^−1^); sodium hydroxide (NaOH, reagent grade, 97%); and glycerol (reagent grade, ≥99.5%) all sourced from Sigma-Aldrich (St. Louis, MO, USA). Additionally, glacial acetic acid (CH_3_COOH, reagent grade 99.5%) was purchased from Fermont (Monterrey, México). Distilled water was used in all preparations. The abbreviations used throughout the manuscript are chitosan (Cs), poly (ethylene glycol) (PEG), glutaraldehyde solution (GA), polyvinylpyrrolidone (PVP) and glycerol (Gly).

### 2.1. Chitosan Production

Chitosan (Cs) was obtained from shrimp waste by alkaline hydrolysis, following the methodology of Sánchez-Duarte et al. [19]. Before the alkaline hydrolysis, shrimp waste was manually separated to remove the legs and tails, leaving only the shells. Subsequently, the shells were boiled in water at 90 °C for 5 min, drained, and dried in an oven at 60 °C. Once dry, they were stored until processing. The process of converting chitin to chitosan was performed in the following stages: (a) Demineralization: dry shells were treated with 1 M HCl for 4 h with constant stirring, then washed with water until a neutral pH was reached; (b) Deproteinization: after the demineralization process, the shell was treated with 4.5% (*w*/*v*) NaOH at 65 °C for 4 h with constant stirring, then washed with water until a neutral pH was reached; (c) Alkaline deacetylation: finally, the shell was treated with 45% (*w*/*v*) NaOH at 110 °C for 2 h with constant stirring to convert chitin into chitosan. The resulting chitosan (Cs) was washed with sufficient water until neutral stage and left to dry at room temperature for subsequent storage.

### 2.2. Chitosan Characterization

#### 2.2.1. Moisture and Ash Content

Following the AOAC [20] method, moisture content was determined by oven drying. A 0.2 g sample of Cs was placed in Petri dishes and dried at 40 ± 1 °C for 24 h. Moisture content was calculated from mass loss; Petri dishes were preheated to constant weight by heating at 110 ± 1 °C for 12 h; then, they were cooled for 20 min and weighed. Ash content was measured gravimetrically: after carbonizing 0.5 g of chitosan in a porcelain crucible; the residues were transferred to a muffle furnace at 600 °C for 12 h. Finally, ash percentage was calculated on a dry basis. All measurements were performed in triplicate.

#### 2.2.2. Molecular Weight and Deacetylation Degree (DD)

Chitosan molecular weight was determined by capillary viscometry using the method described by Solís et al. [21], with minor modifications. In an oil bath at 30 ± 1 °C, the flow times of the solvent (t_0_)—0.3 M acetic acid/0.2 M sodium acetate—and of five chitosan solutions (t_i_) of known concentration (C) were measured in a Ubbelohde viscometer. The relative, specific, and reduced viscosities were calculated as η_rel_ = t_i_/t_0_, η_sp_ = η_rel_ − 1, and η_red_ = η_sp_/C, respectively. The intrinsic viscosity [η] was obtained from the intersection of the linear graph η_red_ versus C. Finally, the average molecular weight (*M_v_*) of chitosan was calculated using the Mark-Houwink equation (Equation (1)):

(1) $$\left[\eta \right]=KM_{v}^{\alpha }\;\to \;M_{v}={\left(\frac{\left[\eta \right]}{K}\right)}^{\frac{1}{\alpha }}$$

For chitosan in HAc/NaAc at 30 °C, *K* = 0.074 mL·g^−1^ and α = 0.76 mL·g^−1^ were used.

The degree of deacetylation (DD) was determined from the FTIR spectrum, which shows the percentage of absorbance (%) as a function of the wavelength (cm^−1^). Based on the intensities of the characteristic bands (1320 and 1420), Equation (2) was applied to calculate the DD, following the method proposed by Brugnerotto et al. [22].

(2) $$\mathrm{DD}=\frac{\left(\left\lceil \frac{-\left(A_{1320}\right)}{\left(A_{1420}\right)}\right\rceil -0.03822\right)}{0.0313}+100$$

#### 2.2.3. Total Nitrogen (Kjeldahl)

The total nitrogen content of chitosan was determined using the Kjeldahl method [20] with slight modifications. A sample of 0.2 g of chitosan, 0.2 g of selenium catalyst, and 8 mL of H_2_SO_4_ were placed in a Kjeldahl flask, and digestion was carried out at 350 ± 1 °C for 6 h. After cooling for 20 min, the digestion was diluted with 10 mL of distilled water, alkalized with 12 mL of 45% NaOH, and subjected to steam distillation at 40 ± 1 °C. The distillate was titrated with 1 N H_2_SO_4_ to determine total nitrogen (%NT) using Equation (3). All determinations were performed in triplicate.

(3) $$\%NT=\frac{\left(V_{H_{2}{SO}_{4}})(N_{H_{2}{SO}_{4}})(0.0111\right)}{m_{sample}}\times 100$$

where V_H2SO4_ = volume of H_2_SO_4_ used in the titration (mL); N_H2SO4_ = acid normality (here, 1 N); m_sample_ = sample mass (g); 0.0111 = conversion factor used for nitrogen in this titration system.

#### 2.2.4. FTIR Spectroscopy

The spectra (400–4000 cm^−1^, 64 scans, resolution 4 cm^−1^) were obtained using a Thermo Scientific Nicolet iS5 spectrophotometer (iD1 transmission), equipped with a fast recovery deuterated triglycine sulfate detector (DTGS, standard) manufactured by Thermo Fisher Scientific (Madison, WI, USA). KBr pellets were prepared by mixing 100 mg of chitosan with 1 g of KBr, grinding the mixture, and pressing it.

### 2.3. Membrane Preparation

The synthesis of chitosan–glutaraldehyde (Cs-GA), chitosan–polyethylene glycol (B2), chitosan–polyvinylpyrrolidone (B5), and chitosan–glycerol (B7) membranes was carried out following the method proposed by Zeng & Fang [23], with minor modifications. For the Cs-GA membrane, a 1.5% (m/v) Cs solution was prepared by dissolving 1.5 g of Cs in 100 mL of 2% acetic acid and then adding 3.85 mL of 0.0107% (*v*/*v*) GA solution. The resulting solution was poured into plastic Petri dishes and placed in an oven (Yamato DX402C, manufactured by Yamato Scientific Co., Ltd., Tokyo, Japan) at 50 ± 1 °C for 16 h to obtain the Cs-GA membranes. Finally, the membranes were neutralized in 2% (*w*/*v*) NaOH at a 1:3 ratio (membrane mass: solution volume) for 30 min, then washed with distilled water to remove residual alkaline residues.

For the preparation of membranes B2, B5, and B7, the corresponding polymer (PEG, PVP, Gly) was added to a 1.5% (m/v) Cs solution in a ratio (2:1) (*w*/*w*) to achieve a concentration of 2.5% (*w*/*v*). Subsequently, 0.0107% GA was added to the chitosan/polymer mixture at a mol: mol ratio. This procedure was carried out under mechanical stirring for 1 h. The final solution was poured into plastic Petri dishes and dried in an oven (Yamato DX402C) at 50 ± 1 °C for 16 h to obtain the membranes. Finally, the membranes were neutralized in 2% (*w*/*v*) NaOH at a 1:3 ratio (membrane mass: solution volume) for 30 min, then thoroughly rinsed with distilled water to remove residual alkaline residues. The compositions used are shown in Table 1.

#### Microporous Formation

Microporous formation in all membranes (Cs-GA, B2, B5, and B7) was performed according to the methods established by Salerno et al. [24] and Zeng et al. [25], with some modifications. The neutralized membrane was kept completely submerged in water in an oil bath at 80–90 °C for 10 h to dissolve the polymer and thus generate the microporous structure. After treatment, the membrane was dried on filter paper to remove excess surface water, then stored in glass Petri dishes for further analysis.

### 2.4. Methods for Characterization

#### 2.4.1. Membrane Thickness

The membrane thickness was measured using a Mitutoyo micrometer (model 7300s, Kawasaki, Japan). For each membrane, measurements were taken at multiple randomly selected points and the average values were then calculated.

#### 2.4.2. Mechanical Stress–Strain Test

The mechanical properties were evaluated according to ASTM D882, following the method proposed by Hernández-Gómez et al. [26] with minor adaptations. Rectangular membrane strips (60 mm × 10 mm) were tested in an Instron 4442 universal testing machine (Instron Corp., USA) at room temperature with a stretching speed of 1 mm·min^−1^. The thickness was measured at the center of each strip with a digital micrometer (accuracy ± 0.001 mm) to calculate the initial cross-sectional area. Adhesive tape was applied to the ends of the sample to prevent tearing during clamping; the initial separation of the jaws was 30 mm. The data were acquired and processed using Instron Series IX software (Compatible with version 8.25, Instron Corporation, Norwood, MA, USA). The stress–strain curve provided the maximum stress (σ_max_), strain at break (ε_max_), and Young’s modulus (E). Four measurements were made per membrane type, and the results are reported as mean ± standard deviation.

#### 2.4.3. Contact Angle

The contact angle was measured using a Dataphysics OCA 15EC goniometer equipped with the accompanying SCA 20 software (Dataphysics Instruments GmbH, Filderstadt, Germany), employing the sessile drop method. Rectangular membrane samples (3 cm × 5 cm) were mounted on the sample platform, and five drops of distilled water (6 µL each) were sequentially deposited at different points on the surface. The images were captured immediately after dosing and analyzed with SCA20 software to obtain the static contact angle; the reported value is the average of five measurements.

#### 2.4.4. Scanning Electron Microscopy (SEM) of Membranes

To study the surface of chitosan-based membranes, samples were metallized with gold in a Quarum Q150R ES vacuum evaporator (Quorum Technologies Ltd., Loughton, UK) for 1 h. Subsequently, a scanning electron microscope (JEOL JSM-7600F, JEOL Ltd., Tokyo, Japan) was used at an acceleration voltage of 5 kV to visualize the membranes obtained.

#### 2.4.5. Atomic Force Microscopy (AFM) Analysis

The 1 cm × 1 cm membrane samples were mounted on a stainless-steel magnetic disc with double-sided adhesive tape and analyzed on a TT-AFM (AFM Workshop, Hilton Head Island, CA, USA). The optical system was focused, and a small tip (radius < 15 nm; monocrystalline silicon; cantilever length 215–235 μm) was brought close to the surface for scanning. For each membrane, images were acquired at multiple distinct locations on the surface using 30 μm × 30 μm scan areas; both height and phase contrast signals were recorded from 2D images using Gwyddion (version 2013; Brno, South Moravia, Czech Republic).

#### 2.4.6. Thermogravimetric Analysis (TGA) and Differential Scanning Calorimetry (DSC)

The thermal stability and degradation of the membranes were measured using a Discovery Series thermogravimetric analyzer (TA Instruments, New Castle, DE, USA). Samples weighing between 6 and 10 mg were heated from 25 to 800 °C at 10 °C·min^−1^ under an N_2_ atmosphere (25 mL·min^−1^). The glass transition temperature (T_g_) was determined using a Discovery Series Differential Scanning Calorimeter (TA Instruments, USA). Samples of between 6 and 10 mg were taken and heated from −50 to 250 °C at 10 °C·min^−1^ under an N_2_ atmosphere (50 mL·min^−1^).

#### 2.4.7. Filtration Tests

The membranes were evaluated in a flat module with an effective area of 1.59 × 10^−3^ m^2^ fed with distilled water from a 20 L externally pressurized tank (maximum operating pressure: 70 psi ≈ 4.83 bar). The permeate was collected on an analytical balance connected to a PC with a data logger, continuously recording the accumulated mass *m*t (g) as a function of time t (s) at room temperature (≈25 °C) [27]. The flux (permeate flow, J) was calculated from the slope dm/dt (g·s^−1^) of the mass-time curve, converting it to volumetric flow rate with the permeate density *ρ* (g·L^−1^) and dividing by the effective membrane area; in units L·m^−2^·h^−1^ (LMH) [28]. Equation (4) was used:

(4) $$J=\frac{1}{A_{m}}\;\frac{1}{\rho }\left(\frac{dm}{dt}\right)\;3600\;\left[LMH\right]$$

where the factor 3600 converts seconds to hours; dm/dt = mass increase rate according to time; *ρ* = permeate density; Am = effective membrane area; *J* = flux.

Additionally, water permeance, L_p_ (LMH·bar^−1^), was reported and calculated from the flux and the applied pressure ΔP (bar) using Equation (5) [29].

(5) $$L_{p}=\frac{J}{\Delta P}\;\left[LMH\;{bar}^{-1}\right]$$

where *J* = flux; ΔP = applied pressure; L*p* = water permeation.

Finally, the total hydraulic resistance R is obtained from Darcy’s relationship between flow, applied pressure, and water viscosity (R = ΔP·µ^−1^·J^−1^). However, since water permeability is defined as L_p_ = J·ΔP^−1^, we have Equation (6).

(6) $$R=\frac{1}{\mu \cdot L_{p}}\;\left[m^{-1}\right]$$

where R = total hydraulic resistance; L_p_ = water permeation; µ = water viscosity (Pa·s).

## 3. Results and Discussion

### 3.1. Characterization of Chitosan: FTIR, Molecular Weight, Total Nitrogen, Moisture, and Ash

**Figure 1:**
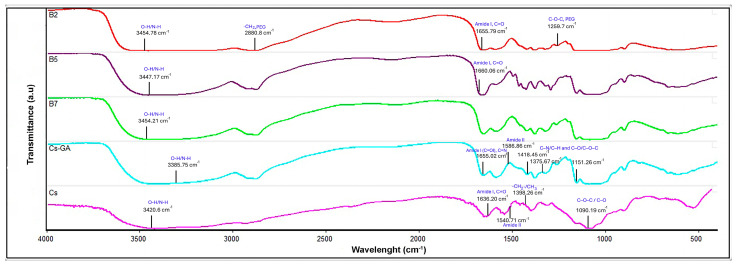
FTIR of chitosan-based membranes. Cs: chitosan, Cs-GA: chitosan–glutaraldehyde, B2: chitosan–polyethylene glycol, B5: chitosan–polyvinylpyrrolidone–polyethylene glycol, and B7: chitosan–glycerol.

The FTIR of Cs extracted from the shrimp shell (Figure 1) shows bands that are typical of polysaccharides. One of these bands, at 3420.6 cm^−1^, is attributed to the stretching vibration of O-H and N-H functional groups [30]. Additionally, bands at 1636.20 and 1540.71 cm^−1^ are found, corresponding to the C=O stretching of amide I and the N-H bending with C-N stretching of amide II, respectively [31,32]. The band at 1398.26 cm^−1^ is attributed to C–H bending vibrations of the –CH_2_ and –CH_3_ groups, while the band at 1090.19 cm^−1^, associated with C–O–C and C–O stretching vibrations of the polysaccharide backbone, confirms the identity of chitosan [33]. The proximate analysis values for chitosan are shown in Table 2. An ash content of 0.17 ± 0.0345% and a moisture content of 5.02 ± 0.2458% are observed, indicating an effective demineralization process and a high degree of purity in the obtained chitosan, as reported by Hosney et al. [34]. Furthermore, the total nitrogen percentage of 6.49 ± 0.4197% indicates an amine-rich chitosan, reflecting a high abundance of -NH_2_ groups [35]. The yield (%) of chitosan from crustaceans is up to a 50%. In this study, the yield was 28% dry mass. Some authors reported this yield using other raw materials and different chemical extraction conditions with values around 23–30% [34,36].

On the other hand, the degree of deacetylation (DD) obtained for chitosan was 93.72%, which is considered high, indicating high purity and quality of the chitosan. According to Nemtsev et al. [37], the degree of deacetylation of chitosan can affect crosslinking formation and solubility, which is considered an important parameter to define the purity of chitosan.

Capillary viscometry determined that chitosan has a molecular weight (Mv) of 456.30 ± 0.99 kDa, which, according to different authors, is classified as a medium molecular weight (100–1000 kDa) [38,39]. This medium molecular weight suggests that chitosan may offer practical processing options due to its viscosity, as well as the crosslinking that may be required to modify its mechanical properties in membrane fabrication [40].

### 3.2. Membrane Characterization

#### 3.2.1. FTIR

Figure 1 shows the FTIR spectra of Cs and the Cs-GA, B2, B5, and B7 membranes. Comparing the FTIR spectra of Cs and Cs-GA reveals that the fundamental chemical structure of chitosan is conserved, but modifications are observed in several key bands associated with glutaraldehyde crosslinking. In Cs-GA, the broad O–H/N–H stretching band is centered at 3385.75 cm^−1^, remaining within the typical range reported for Cs. The most significant change is observed in the 1700–1500 cm^−1^ region: the amide I band shifts to 1655.02 cm^−1^, which is interpreted as the overlap of the amide’s C=O bond with the new C=N band (Schiff base) formed by the Cs–GA crosslinking. This result is consistent with that reported by Galan et al. [41] in chitosan beads crosslinked with glutaraldehyde. Similarly, in Cs, the 1540.71 cm^−1^ band shows a shift in the range associated with N–H bending, reaching 1586.85 cm^−1^ in Cs-GA [42]. Finally, the bands at 1418.45, 1375.67, and 1151 cm^−1^ of Cs-GA remain within the typical region corresponding to C–N/C–H and C–O/C–O–C vibrations of polysaccharides [43], although with slight changes in position and intensity, indicating that the chitosan network is maintained. However, glutaraldehyde crosslinking alters its chemical environment.

In membranes (B2, B5, and B7), the O–H/N–H band shifts towards higher frequencies (3443–3454 cm^−1^) and exhibits less width and intensity compared to Cs, suggesting partial consumption of –NH_2_ and –OH groups due to the formation of imine and acetal bonds during crosslinking with glutaraldehyde [44,45]. Although the glutaraldehyde concentration was very low (0.0107%), the observed shifts confirm the effectiveness of crosslinking, even in the absence of a defined C=N band, which is usually overlapped or undetectable at low concentrations [46,47].

In membrane B2, additional signals appear at 2880 cm^−1^ (C–H stretching of –CH_2_–) and 1259 cm^−1^, attributable to the methylene and ether groups of the incorporated PEG [48]. In membrane B5, a single, intense peak is observed at 1660 cm^−1^, replacing the separate amide I and II bands of Cs, a typical behavior when PVP is incorporated due to the overlap of the carbonyl group of the pyrrolidone ring (1660–1680 cm^−1^) with amide I of chitosan [49,50,51].

In membrane B7, the O–H/N–H band moves slightly to 3454.21 cm^−1^, but the Cs-GA spectral pattern stays the same. This behavior confirms that, when Gly is incorporated as a plasticizer, no new covalent bonds are formed; instead, hydrogen bonding interactions predominate between the hydroxyl groups of glycerol and the –OH/–NH_2_ groups of Cs [52,53]. In summary, the FTIR spectra confirm the effective crosslinking of chitosan with glutaraldehyde in Cs-GA, B2, B5, and B7, as well as the successful incorporation of PEG and PVP in B2 and B5, and the minimal chemical modification of Cs in the presence of Gly (B7).

#### 3.2.2. Morphology and Topography (SEM and AFM)

**Figure 2:**
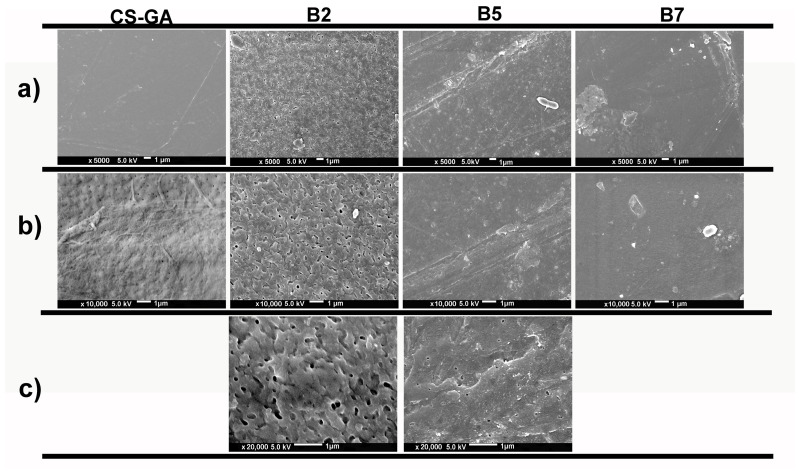
SEM micrograph of chitosan-based membranes at (**a**) 5000×, (**b**) 10,000×, and (**c**) 20,000×. Cs-GA: chitosan–glutaraldehyde, B2: chitosan–polyethylene glycol, B5: chitosan–polyvinylpyrrolidone–polyethylene glycol, and B7: chitosan–glycerol.

Figure 2a displays SEM micrographs of the various membranes at a magnification of 5000×. Cs-GA exhibits a compact and continuous surface with little to no visible porosity, a result very similar to that reported by Cui et al. [54]. In contrast, membrane B2 shows a rougher, more heterogeneous texture, with surface depressions and deep cavities characteristic of porous materials, consistent with the findings of Zeng & Fang [23]. Membrane B5 exhibits an intermediate morphology, with interconnected pores and grooves forming an open network that may facilitate fluid displacement, as reported by Shirazi et al. [55]. Finally, membrane B7 shows a smoother surface, compared to membranes B2 and B5, with apparent polymeric aggregates on the surface, making it difficult to distinguish between the Cs-GA membrane, this could be attributed to the plasticizing effect of glycerol, which tends to smooth surfaces, a behavior consistent with that described by Liu et al. [56] and Pinto et al. [57].

Figure 2b presents SEM micrographs of the same membranes at 10,000× magnification. The Cs-GA membrane exhibits fine undulations and microreliefs, without evident pore openings; in the upper zone, scattered depressions are visible, possibly associated with the collapse of microbubbles during the membrane formation process, as noted by Galiano et al. [58]. The B2 membrane has pores that show convergence and connectivity with neighboring cavities, forming continuous channels across its entire surface. In contrast, the B5 membrane exhibits only a limited number of sparsely distributed pores, which may be easily mistaken for irregular polymeric clusters. The B7 membrane exhibits fine micro-wrinkles; however, polymeric aggregates predominate on the observed surface.

The high-resolution SEM micrographs (Figure 2c) show that membranes B2 and B5 are the most promising. Membrane B2 exhibits a high density of submicrometer cavities with sharp edges and signs of fusion and connectivity between gaps, forming an open and continuous structure that may be favorable for fluid transport. Although membrane B5 has a more compact surface compared to B2, it exhibits well-distributed, isolated pores and fine grooves, resulting in moderate roughness without extensive discontinuities. Discrete inclusions and gently differentiated domains are also observed, suggesting a stable microstructure. In general, B2 has more interconnected pores, while B5 has a uniform matrix with controlled porosity. Both microstructures are considered beneficial for filtration processes, as reported for Cs-PEG membranes (B2) [59,60,61].

**Figure 3:**
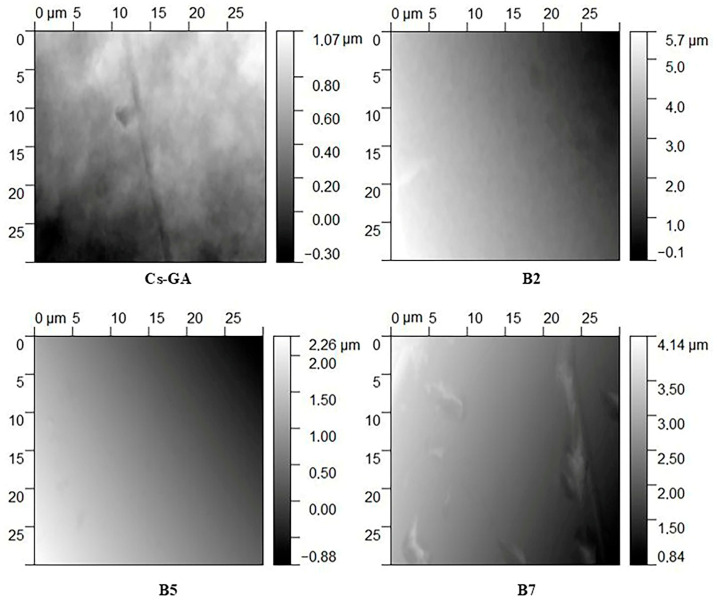
AFM topographies (30 µm × 30 µm) of the chitosan-based membranes. Cs-GA: chitosan–glutaraldehyde, B2: chitosan–polyethylene glycol, B5: chitosan–polyvinylpyrrolidone–polyethylene glycol, and B7: chitosan–glycerol.

The AFM topography (Figure 3) confirms the trends observed by SEM: membranes containing PEG exhibit more corrugated, heterogeneous surfaces, consistent with the findings of He et al. [59]. Cs-GA exhibits the lowest roughness amplitude and a compact, continuous topography. Membrane B2 displays the greatest corrugation, with persistent micrometric valleys even after leveling, confirming its surface heterogeneity. Membrane B5 exhibits intermediate roughness, attributed to the PEG-PVP combination, where PEG induces a more pronounced relief than Cs-GA, but less than B2. Finally, membrane B7 exhibits broad undulations and elongated ridges, consistent with the plasticizing effect of Gly reported by Galo et al. [62].

#### 3.2.3. Thickness and Hydrophilic Character of the Membranes (Contact Angle)

Table 3 shows the thickness and contact angle results for each membrane. The thicknesses range from approximately 300 to approximately 123 μm, with the modified membranes being thinner than pure chitosan. Wanichapichart & Yu [63] indicate that chitosan membranes used for filtration typically have a thickness of 40–80 μm. The pure chitosan membranes developed in this study are thicker than 300 μm. This considerably greater value is attributed to the higher molecular weight of the chitosan used (Mv ≈ 456.30 kDa). Several authors have reported that a higher molecular weight increases the viscosity of the film-forming solution, thereby favoring the formation of thicker, mechanically stronger films [40,64,65]. Al-Hobaib et al. [66] report that commercial reverse osmosis membranes have a total thickness of approximately 130 to 250 μm, corresponding to three structural layers (bottom, middle, and top). These values are comparable to those obtained for membranes B2 and B5. However, in our study, the membranes lack additional layers or any additional support, so the reported thickness corresponds to a single functional layer. In membrane B7, glycerol acts as a plasticizer, increasing the mobility of the chitosan chains, as described by Nwaka et al. [67]. Similarly, Rivero et al. [68] indicate that glycerol in the chitosan polymer matrix increases molecular mobility and promotes the rearrangement of the polymer network, which could explain its reduced thickness. This decrease is because greater mobility promotes more intense compaction during drying, resulting in a lower thickness than in B2 and B5.

Analysis of the hydrophilic-hydrophobic character revealed that all membranes exhibited contact angles below 90°, indicating hydrophilic surfaces, as reported by Kertész et al. [69]. However, differences were observed among the membrane values (Table 3). The Cs-GA membrane had the largest contact angle (89.39 ± 3.65172°), a value very close to that of hydrophobic surfaces (θ ≥ 90°), according to Chen et al. [70]. Membranes B2 and B5 presented the lowest angles, which agrees with the AFM and SEM results, where more corrugated surfaces with connected domains were observed, favoring the spreading of the water droplet across the surface. Taken together, their lower thickness and greater hydrophilicity in B2 and B5 suggest that these membranes are more favorable for more efficient water transport, a topic discussed in more detail in the filtration tests section.

#### 3.2.4. Mechanical Properties (Maximum Stress, Strain at Break, Young’s Modulus)

**Figure 4:**
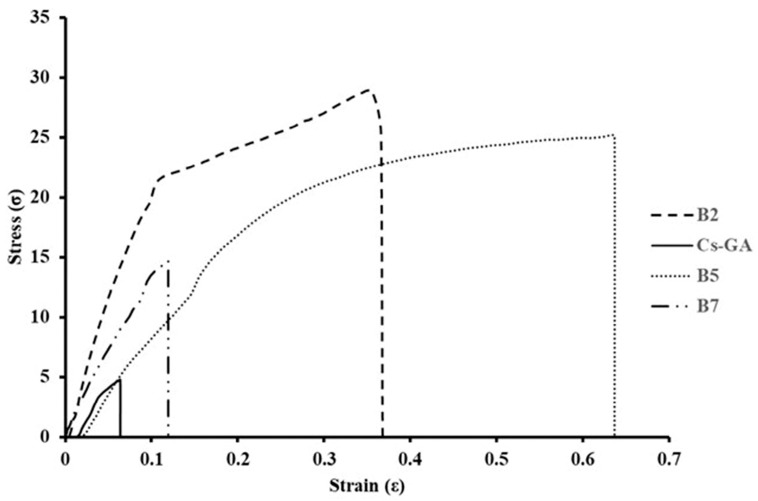
Stress–strain curves for the different membranes. Cs-GA: chitosan–glutaraldehyde, B2: chitosan–polyethylene glycol, B5: chitosan–polyvinylpyrrolidone–polyethylene glycol, and B7: chitosan–glycerol.

Table 4 shows the mechanical property values. The Cs-GA membrane exhibited the most brittle behavior, with a σ_max_ of 2.612 ± 1.479 MPa and a strain at break of 9.10 ± 0.0459%, reflected in a short stress–strain curve with a low slope (Figure 4). This data indicates a rigid matrix that, despite being cross-linked with GA, is fragile and has low mechanical strength, in accordance with the findings of Silva et al. [71], and may be susceptible to failure under relatively low stresses during handling or operation.

The incorporation of PEG into the B2 membrane resulted in the greatest increase in strength and stiffness. The maximum stress (σ_max_) increases to 24.59 ± 2.69 MPa and Young’s modulus reaches 0.179 ± 0.070 GPa, while the strain at break is 16.99 ± 9.79%. In Figure 4, the B2 curve shows a steep initial slope and a moderate deformation interval before failure. This behavior is consistent with that reported by He et al. [59] for Cs-PEG systems and with the more open microstructure observed by SEM and AFM.

The B5 membrane exhibited the highest deformation capacity, with a strain at break of 27.10 ± 8.38%, a σ_max_ of 15.48 ± 3.70 MPa, and a Young’s modulus of 0.058 ± 0.006 GPa. In Figure 4, the B5 curve exhibits a very long deformation zone (plastic zone), indicating high toughness. This performance relates to the previously observed hybrid morphology. B5 has a more compact surface than B2, yet it has connected pores and grooves that form a well-opened network. These results are consistent with those reported by He et al. [59] and Sarhan [72] for Cs-PEG blends. They are also consistent with the work of Raza et al. [73], which indicates that Cs-PVP blends exhibit improved mechanical properties, excellent compatibility, and stability, attributed to the interactions between the two chains.

Finally, membrane B7 shows that σ_max_ is 9.36 ± 5.14 MPa, strain at break is 11.29 ± 3.62%, and Young’s modulus is 0.086 ± 0.031 GPa. Its stress–strain curve (Figure 4) shows an intermediate initial slope and a relatively limited deformation zone. These mechanical results are consistent with the smoother topography and lower surface porosity of membrane B7 (Figure 2 and Figure 3), attributable to the plasticizing effect of Gly, which smoothes the surface and promotes a more compact rearrangement of the polymer network. Membrane B7 is better than Cs-GA, but it does not have the balance of strength and ductility as B2 and B5. Leceta et al. [74] reported gly-plasticized chitosan films with maximum stress of 55–62 MPa and elongations near 0.5, characteristics of relatively rigid and brittle materials. The B7 membrane has lower strength but a significantly higher deformation capacity prior to failure. From a membrane engineering perspective, parameters such as tensile strength and strain at break are decisive for mechanical stability under pressure gradients [75]. In this sense, the greater deformation observed in B7 before breaking suggests mechanical behavior compatible with its potential use in water filtration applications.

#### 3.2.5. Thermal Behavior (TGA and DSC)

**Figure 5:**
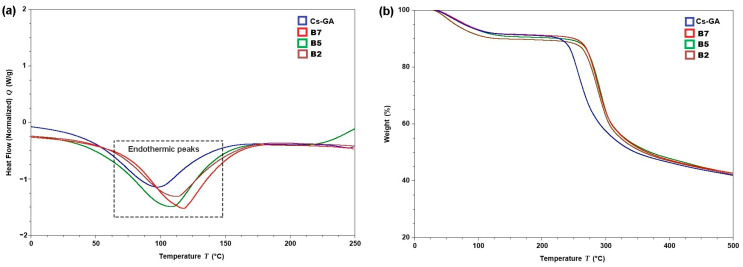
Thermograms of the different membranes: (**a**) DSC and (**b**) TGA. Cs-GA: chitosan–glutaraldehyde, B2: chitosan–polyethylene glycol, B5: chitosan–polyvinylpyrrolidone–polyethylene glycol, and B7: chitosan–glycerol.

Figure 5a shows an endothermic peak in all thermograms; in the case of Cs-GA, the decrease in heat flow rate (W/g) begins at 10 °C, followed by B7, B5, and B2, which begin to lose heat at 25 °C. This is attributed to the heat resistance provided by each crosslink or graft to the Cs-GA polymer network, which prolongs the loss of water and other volatile compounds per unit mass [76]. The maximum endotherm temperature for B5 was 106 °C, for B2 it was 112.5 °C, and for B7 it was 118 °C; for Cs-GA, it was below the values observed in the membrane (<100 °C). In the case of the B5 membrane, there is an interaction between the hydroxyl groups of Cs and the carbonyl group of PVP. Sizílio et al. [77] suggest that the pyrrolidone rings in PVP contain a carbonyl group that accepts protons, while chitosan has hydroxyl and amino groups as side groups; therefore, hydrogen bonding can occur between these two chemical groups.

In the case of TGA (Figure 5b), an initial phase of weight loss was observed in the Cs-GA membrane at temperatures below 225 °C, while for membranes B2, B5, and B7, this loss was detected at temperatures below 275 °C; at these temperatures, only the loss of water molecules is indicated [78,79]. Likewise, a second stage of weight loss is observed above the aforementioned temperatures (225 °C for Cs-GA and 275 °C for membranes with B2, B5, and B7 grafts). In this stage, degradation is more intense due to thermal degradation of chitosan, leading to the loss of functional groups (amino groups, hydroxyl groups, etc.); however, membranes with a cross-linking agent and/or grafting exhibit higher degradation temperatures [80]. The percentage of normalized weight loss recorded in the second stage indicates that Cs loses mass more quickly and with a lower percentage. Cs-GA lost 58.48% of its mass, while the rest of the membranes behaved very similarly, recording losses of 62.99% for B7, 67.73% for B5, and 70.748% for B2. The third and final stage of decomposition starts at 262 °C, 288.91 °C, 290.59 °C, and 294.32 °C for Cs-GA, B7, B5, and B2, respectively. This is in line with the mechanical test results for membranes that use PEG.

### 3.3. Membrane Performance in Filtration Tests

Table 5 presents the flux (*J*), water permeance (L_p_), and total hydraulic resistance (R) values of the membranes evaluated under filtration by distilled water at 4.83 bar. Membrane B2 showed superior performance compared to the other membranes, with much higher flux and water permeance values and a much lower hydraulic resistance. The Cs-GA, B5, and B7 membranes exhibit flux values of 24.10, 40.43, and 24.77 LMH, respectively, and permeance values that do not exceed 10 LMH·bar^−1^, while the B2 membrane reaches 2222.70 LMH and a permeance of 460.19 LMH·bar^−1^. Consistently, the hydraulic resistance of Cs-GA, B5, and B7 is in the range of 4.15 × 10^13^ to 7.89 × 10^13^ m^−1^, compared to the value of 8.79 × 10^11^ m^−1^ for B2. Although the *J* and L_p_ values of B2 are much higher than those of Cs-GA, B5, and B7, its permeance is still lower than that of some commercial ultrafiltration membranes used for seawater pretreatment. Cordier et al. [81] reported an initial permeability of approximately 880 LMH·bar^−1^ and a membrane resistance of 4.1 × 10^11^ m^−1^ for a polysulfone hollow fiber membrane used in seawater ultrafiltration, operating with nearly constant fluxes of 60–80 LMH. The results of this study show that water passes through the B2 membrane much more easily than through the other membranes evaluated; this implies that lower flux and permeance values correspond to higher hydraulic resistance.

On the other hand, the Cs-GA, B5, and B7 membranes exhibit much lower filtration performance. The B5 membrane, which has a mixture of PEG and PVP in the Cs chain, reports the highest flux within this group (40.43 LMH), but its permeability (8.37 LMH·bar^−1^) and hydraulic resistance (4.83 × 10^13^ m^−1^) are very similar to those of Cs-GA. In other words, although B5 allows slightly more water to pass through than the Cs-GA membrane, the overall resistance to flow remains high. This result is consistent with the idea that the combination of PEG and PVP generates a corrugated surface but with a more homogeneous, relatively compact matrix that partly limits the paths available for water to pass through. In the case of the B7 membrane, the flux (24.77 LMH) is similar to that of Cs-GA, but the permeance is the lowest compared to the other results (5.13 LMH·bar^−1^), and the hydraulic resistance is the highest at 7.89 × 10^13^ m^−1^. This observation suggests that, although B7 is a thin membrane, the presence of Gly and the rearrangement of the polymer network during drying result in a less porous structure, making it more difficult for water to pass through.

The relatively low flux values and high hydraulic resistances observed for Cs-GA, B5, and B7 (flux in the range of 24–40 LMH and R of the order of 10^13^ m^−1^) are numerically comparable to values reported for ultrafiltration membranes subjected to high salinity and fouling conditions; Scelfo et al. [82] describe permeance values of 7.4–17 LMH·bar^−1^ in their study. This suggests that these chitosan-based membranes exhibit very high hydraulic resistance, even during distilled water filtration.

## 4. Conclusions

The results of the present study show that the chitosan obtained allowed for the production of stable membranes, whose modification with PEG, PVP, and Gly and cross-linking with GA produced a dense and compact structure (Cs-GA) with more open, corrugated surfaces (B2 and B5) or thinner but compact surfaces (B7). Additionally, B2 and B5 exhibited a higher degree of hydrophilicity. These structural changes were reflected in the mechanical properties, with membranes B2 and B5 exhibiting higher maximum stress (σ_max_) and greater strain at break (ε_max_) than Cs-GA, indicating better tensile performance. In water filtration tests, the B2 membrane had the highest flow and permeability and the lowest hydraulic resistance, consistent with its lower thickness, higher porosity, and hydrophilic nature. At the same time, B5 showed intermediate performance, with moderate flow but a more compact and mechanically stable structure. However, Cs-GA and B7 exhibited higher hydraulic resistances and more limited water transport. In conclusion, these results indicate that membranes B2 and B5 are the most promising for their use in water filtration processes, since they combine hydrophilicity, adequate mechanical and thermal properties, and superior filtration performance to Cs-GA. Therefore, their evaluation is recommended in future work with real water flows and under prolonged operating conditions.

## Figures and Tables

**Table 1:** Formulations used for the preparation of membranes.

Membrane	Cs (g)	PEG (g)	PVP (g)	Gly (g)	Solution GA (mL)
Cs-GA	1.50	—	—	—	3.850
B2	1.50	0.75	—	—	3.850
B5	1.50	0.35	0.35	—	3.850
B7	1.50	—	—	0.75	3.850

Cs = chitosan; PEG = poly (ethylene glycol); PVP = polyvinylpyrrolidone; Gly = glycerol; GA = glutaraldehyde.

**Table 2:** Proximate analysis of chitosan.

Parameters	Results (%)
Moisture	5.02 ± 0.2458
Total Nitrogen	6.49 ± 0.4197
Ash Content	0.17 ± 0.0345

The reported values are the average of n = 3 ± standard deviation.

**Table 3:** Thickness and contact angle of the membranes studied.

Membrane	Thickness (μm)	Contact Angle (°)
Cs-GA	300 ± 10.12 ^1^	89.39 ± 3.6517 ^2^
B2	207 ± 40.40 ^1^	56.68 ± 1.4249 ^2^
B5	190 ± 26.85 ^1^	58.94 ± 4.7231 ^2^
B7	123 ± 15.37 ^1^	65.20 ± 5.1517 ^2^

Cs-GA: chitosan–glutaraldehyde, B2: chitosan–polyethylene glycol, B5: chitosan–polyvinylpyrrolidone–polyethylene glycol, and B7: chitosan–glycerol. ^1^ The reported values are the mean of n = 3 ± standard deviation. ^2^ The reported values are the mean of n = 5 ± standard deviation.

**Table 4:** Mechanical properties of the membranes (σ_max_, ε_max_, and Young’s modulus).

Membrane	Maximum Stress (σ_max_) (MPa)	Strain at Break (ε_max_) (%)	Young’s Modulus (GPa)
Cs-GA	2.612 ± 1.479	9.10 ± 0.0459	0.037 ± 0.028
B2	24.589 ± 2.690	16.99 ± 9.79	0.179 ± 0.070
B5	15.478 ± 3.705	27.10 ± 8.38	0.058 ± 0.006
B7	9.361 ± 5.144	11.29 ± 3.624	0.086 ± 0.031

Cs-GA: chitosan–glutaraldehyde, B2: chitosan–polyethylene glycol, B5: chitosan–polyvinylpyrrolidone, and B7: chitosan–glycerol. The reported values correspond to the average ± standard deviation of n = 3.

**Table 5:** Membrane performance in water filtration tests.

Membrane	Flux (*J*) (LMH)	Permeance (L_p_) (LMH/bar)	Hydraulic Resistance (R) (m^−1^)
Cs-GA	24.0988	9.7513	4.1481 × 1013
B2	2222.70	460.187	8.7900 × 1011
B5	40.4345	8.3715	4.8317 × 1013
B7	24.7701	5.1284	7.8873 × 1013

Cs-GA: chitosan–glutaraldehyde, B2: chitosan–polyethylene glycol, B5: chitosan–polyvinylpyrrolidone–polyethylene glycol, and B7: chitosan–glycerol.

## Data Availability

The original contributions presented in this study are included in the article. Further inquiries can be directed to the corresponding author.

## Abbreviations

The following abbreviations are used in this manuscript:

Cs	Chitosan
PEG	Polyethylene glycol
PVP	Polyvinylpyrrolidone
Gly	Glycerol
GA	Glutaraldehyde
FTIR	Fourier-transform infrared spectroscopy
SEM	Scanning electron microscopy
AFM	Atomic force microscopy
TGA	Thermogravimetric analysis
DSC	Differential scanning calorimetry
LMH	Liters per square meter per hour (LMH)
LMH·bar^−1^	Liters per square meter per hour per bar (LMH·bar^−1^)
*J*	Permeate flux (J)
L_p_	Water permeance (L_p_)
R	Total hydraulic resistance (R)
kDa	Kilodaltons
M_w_	Molecular weight (Mw)
η	Intrinsic viscosity (η)
M_v_	Viscosity-average molecular weight (Mv)
η_rel_	Relative viscosity (η_rel_)
η_sp_	Specific viscosity (η_sp_)
η_red_	Reduced viscosity (η_red_)
%NT	Total nitrogen percentage (%NT)
T_g_	Glass transition temperature (T_g_)
ΔP	Applied pressure (ΔP)
Cs-GA	Chitosan–glutaraldehyde
B2	Chitosan–polyethylene glycol
B5	Chitosan–polyvinylpyrrolidone–polyethylene glycol
B7	Chitosan–glycerol
